# Climate change favors expansion of three *Eucalyptus* species in China

**DOI:** 10.3389/fpls.2024.1443134

**Published:** 2024-10-11

**Authors:** Xinjie Mao, Huisen Zheng, Guihua Luo, Songkai Liao, Ronghao Wang, Ming Tang, Hui Chen

**Affiliations:** Guangdong Laboratory for Lingnan Modern Agriculture, State Key Laboratory of Conservation and Utilization of Subtropical Agro-Bioresources, College of Forestry and Landscape Architecture, South China Agricultural University, Guangzhou, China

**Keywords:** species distribution models, timber trees, maxent, potential distribution, suitable range

## Abstract

*Eucalyptus* has become one of the most widely planted species in tropical and subtropical regions in China, with important economic, ecological, and social values. However, it is currently unclear how climate change will affect different *Eucalyptus* species. Therefore, it is urgent to investigate the potential distribution and dynamics of *Eucalyptus* under current and future climate scenarios. In this study, we analyzed the potential distribution patterns of the three main *Eucalyptus* species (*Eucalyptus grandis*, *Eucalyptus urophylla*, and *Eucalyptus tereticornis*) under current and future climatic conditions (2041-2060 and 2061-2080) using the optimized MaxEnt model, which integrates a variety of environmental data including climate, topography, soil, and human influence. We also identified the main factors affecting the potential distributions of the three main *Eucalyptus* species. The model indicated that *E. grandis* exhibited heightened sensitivity to the mean temperature of the coldest quarter (7.0-20.0 °C) and annual mean temperature (11.9-24.2 °C), whereas *E. urophylla* displayed heightened sensitivity to precipitation of the warmest quarter (272-1694 mm) and annual precipitation (812-2624 mm). Conversely, *E. tereticornis* demonstrated heightened sensitivity to annual mean temperature (12.7-24.5 °C) and temperature seasonality (63.8-598.9). Under the current climate, *E. tereticornis* had the widest suitable distribution area (124.91 × 10^4^ km²), followed by *E. grandis* (124.89 × 10^4^ km²) and *E. urophylla* (119.81 × 10^4^ km²). Under future climate change scenarios, the suitable ranges of *E. grandis*, *E. urophylla* and *E. tereticornis* will continue to expand. This study highlights the importance of climate change in *Eucalyptus* distribution and provides quantified potential distribution maps for three *Eucalyptus* species under current and future climate conditions in China. This research offers valuable scientific insights pertinent to the management and rational site selection for *Eucalyptus* plantations.

## 1 Introduction

China has the largest forest plantation area in the world, with a total area of 79.54 × 10^6^ hm^2^ (Du et al., 2020; Zhao et al., 2021a). Forest plantations are important components of forest ecosystems, providing resources for human needs and contributing to timber production, environmental improvement, and climate change mitigation efforts (Payn et al., 2015; Gabira et al., 2023). *Eucalyptus* is significant forest plantation species and have the largest plantation area in the world, with 25 million hectares planted (Elli et al., 2020; Martins et al., 2022). *Eucalyptus* plants exhibit rapid growth, high stress resistance, tolerance to poor soils, and a trunk shape ideal for timber production, rendering them suitable for diverse applications (Booth, 2013; Zhu et al., 2020a; Yang et al., 2023). In southern China, *Eucalyptus* have been extensively planted and currently occupy approximately 5.4 million hectares (Zhang and Wang, 2021).

The selection of *Eucalyptus* species for plantation in China, notably encompassing *Eucalyptus urophylla*, *Eucalyptus grandis*, *Eucalyptus dunnii*, *Eucalyptus tereticornis*, and their hybrids (Arnold et al., 2020; Zhang and Wang, 2021). It is important to select stable plantations with suitable climatic conditions for different species of *Eucalyptus* (Booth et al., 2017; Chen et al., 2022; Ouyang et al., 2022). Furthermore, global climate change will significantly affect the suitability of *Eucalyptus* cultivation in various regions in the long term (Booth, 2013; Aspinwall et al., 2016; Drake et al., 2017; Florêncio et al., 2022). Climate constitutes a primary determinant governing the spatial distribution patterns of plant species, exerting profound impacts on their growth and survival (Cetin et al., 2023). Presently, escalating temperatures and alterations in precipitation patterns are instigating transformations in the global environment, thereby exerting multifaceted influences on ecosystems worldwide (Clark, 2004; Hamann et al., 2021a; Singh et al., 2023). These changes reverberate through various dimensions, impinging upon the morphology, physiology, and distributional range of species, individuals, and populations (Kumarathunge et al., 2020; Everingham et al., 2021; Zu et al., 2022; Wang et al., 2023a). Therefore, it is significant to investigate how *Eucalyptus* species respond to climate change. This information is crucial for their cultivation and energy development in China.

Species distribution models (SDMs) based on ecological niche theory are commonly used to predict the potential distribution and habitat suitability of species (Elith and Leathwick, 2009; Early et al., 2022; Li et al., 2023). In the case of plants, SDMs have been applied to predict the potential distribution of invasive, endangered, and medicinal plants, as well as the suitability of crop planting (Yang et al., 2022; Boral and Moktan, 2024). Currently, commonly used species distribution models include random forest, Genetic Algorithm (GARP), CLIMEX and MaxEnt (Stockwell, 1999; Cutler et al., 2007; Phillips and Dudík, 2008; Kriticos et al., 2015). Several model comparison studies have shown that the MaxEnt model, based on the principle of maximum entropy, typically has superior ability to handle pseudoabsence data compared to other SDMs (Velasco and González-Salazar, 2019; Kaky et al., 2020). In addition, Maxent has been reported to perform well even when the distribution of species is poorly documented (Pearson et al., 2007). MaxEnt has been employed in various studies concerning the assessment of climate suitability for introduced tree species (Puchałka et al., 2021; Sychrová et al., 2022; Puchałka et al., 2023). In recent years, there are many researchers have studied the potential distribution of *Eucalyptus* species around the world (Booth et al., 2017; Shabani et al., 2017; Resquin et al., 2020). In China, Ouyang et al. (2022) predicted the potential distribution of *E. grandis* based on the Coupled Model Intercomparison Project Phase 5 (CMIP5) climate dataset. *Eucalyptus grandis* expands its potential range in China under both CMIP5 future climate scenarios, and the area of expansion in 2041-260 is more variable than the area of expansion in 2061-2080.

However, the current CMIP6 models differ from CMIP5 due to stronger projected warming, higher climate sensitivity in the new generation of models, and updated specifications for concentration pathways, emissions, and socio-economic development. Compared to the CMIP5 model, CMIP6 provided higher accuracy and reliability in predicting potential areas of suitability for species (Gidden et al., 2019; Gülçin et al., 2021). To date, CMIP6 climate data have not been applied in China to predict the suitability of *Eucalyptus* plantations, and existing studies have not integrated multiple factors to comprehensively analyze the suitability of *Eucalyptus* planting under future climate change scenarios. Consequently, there remains a gap in understanding the potential responses of *Eucalyptus* plantations to future climate changes in China.

In this study, the optimized Maxent model was used to predict the potentially suitable areas for three *Eucalyptus* species, namely, *E. grandis*, *E. urophylla*, and *E. tereticornis* planted in China under current and future climate scenarios. The objectives of this study were to: (1) assess the suitability of *E. grandis*, *E. urophylla* and *E. tereticornis* for planting in different regions of China; (2) identify the main environmental variables limiting their potential distribution; and (3) analyze the regional changes in the potential suitability of these three *Eucalyptus* species in China under future climate change scenarios.

We hypothesized that (i) *E. tereticornis* would have the largest potential suitable area (Booth and Pryor, 1991), (ii) the potential suitable areas of the three species would expand under future climatic conditions, and (iii) the change in the distribution of the optimal climatic conditions would be greater in 2041-2060 than in 2061-2080. The forecasted outcomes of this study bear significant implications for sustaining the stability of plantation forest ecosystems in China. Furthermore, they furnish a scientific foundation for the planning and management strategies concerning *Eucalyptus*.

## 2 Materials and methods

### 2.1 Studied species

*Eucalyptus grandis*, a species native to Australia and introduced to China in the 1960s, has become one of the major fast-growing species used for afforestation of arable lands in southwestern China (Zhou et al., 2020; Wang et al., 2023b). This species typically grows as a tall, straight forest tree, reaching heights of around 50 meters, with a diameter at breast height (DBH) of 1.2 to 2 meters. The wood of *E. grandis* is relatively soft, with a straight grain, making it suitable for processing into various products, including artificial boards, pulp, mine props, and firewood. Additionally, it is used as a protective forest species and for landscaping purposes, offering significant economic benefits (Booth, 2013; Bandara and Arnold, 2017).

*Eucalyptus urophylla*, native to the Indonesian archipelago and among the most widely distributed eucalypt species in terms of altitude, was introduced to China in 1976 and is now cultivated in Guangdong, Guangxi, and Hainan provinces (Eldridge et al., 1994; Kien et al., 2009). *Eucalyptus urophylla* is an evergreen broad-leaved tree, reaching heights of up to 60 meters and a DBH of 2.0 meters. It has become a major species for short-rotation industrial timber plantations in southern China, particularly for the pulp and artificial board industries (Dlamini et al., 2017).

*Eucalyptus tereticornis*, known for its drought tolerance and salt-alkali resistance, is naturally distributed along the eastern coastal regions of Australia (Luo et al., 2014). *Eucalyptus tereticornis* is a tree that typically grows to a height of 20-50 m and has a girth of up to 2 m DBH (Boland et al., 2006). This species is highly valued for solid wood and industrial timber applications, primarily used in construction, bridge building, and shipbuilding (Ginwal, 2009). *Eucalyptus tereticornis* was first introduced to China in 1890 (Yang, 2019).

### 2.2 Occurrence records

**Figure 1:**
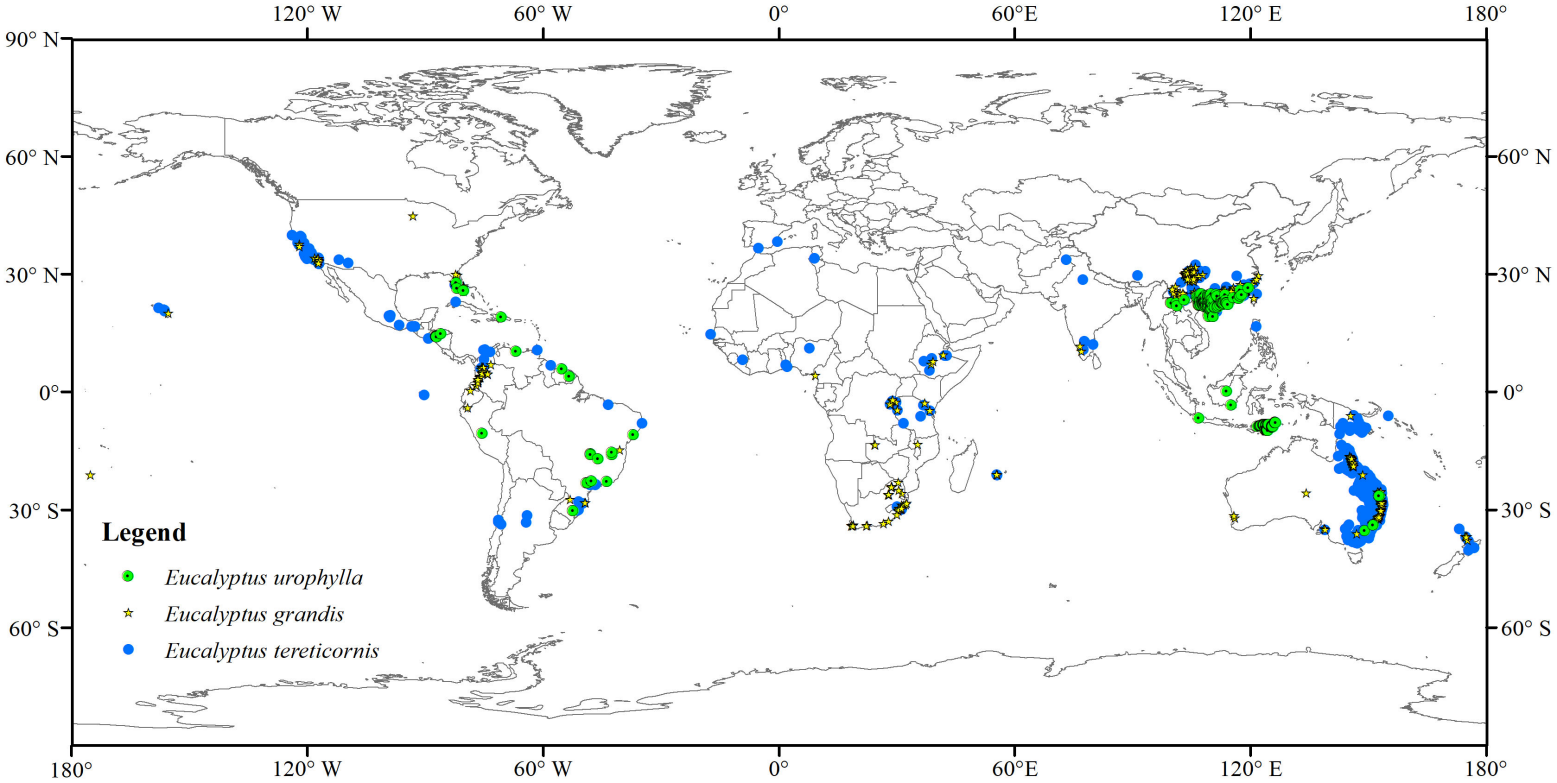
The occurrence records of *Eucalyptus grandis, E. urophylla,* and *E. tereticornis*.

In this study, we utilized global occurrence records to predict the potential distribution of three species (i.e., *E. grandis*, *E. urophylla*, and *E. tereticornis*) in China. Incorporating all available distribution records, encompassing both native and introduced occurrences, serves to diminish model uncertainty and bolster its reliability (Jiménez-Valverde et al., 2011; Shabani and Kumar, 2015). The distribution data of the three species were obtained from Chinese Virtual Herbarium (https://www.cvh.ac.cn) and Global Biodiversity Information Facility (https://www.gbif.org). Furthermore, distribution data were sourced from pertinent literature through thesis databases such as CNKI (China national knowledge infrastructure) and Web of Science (Payn et al., 2008; Song et al., 2016; Zhu et al., 2020b; Chen et al., 2021). All data points were carefully checked, with duplicates and erroneous records removed. To reduce sampling bias and ensure equal representation of particular grid cells, we used SDMtoolbox to retain only one species occurrence point within every 2.5 arc-minutes (Brown, 2014). Finally, a total of 344 (224 from GBIF, 14 from CVH and 106 from literature data), 217 (62 from GBIF, 3 from CVH and 152 from literature data), and 2645 (2610 from GBIF, 18 from CVH and 17 from literature data) distribution records of *E. grandis*, *E. urophylla*, and *E. tereticornis* were obtained, respectively (**Figure 1**).

### 2.3 Collection and screening of environmental variables

A total of 27 environmental factors were used in the projection, consisting of 19 bioclimatic factors, 3 topographical factors, 4 soil factors, and 1 human influence factor (**Supplementary Table S1**). The current climate data (1970-2000), obtained from the WorldClim database, have the advantage of being ecologically significant and high resolution. It encompasses 19 bioclimatic variables that depict changes in temperature and precipitation under current climatic conditions, one of the most comprehensive climate data available for species distribution modeling. However, to gain a comprehensive understanding of the environmental factors influencing the distribution of *Eucalyptus*, we also incorporated variables related to soil, topography, and human impact variables. Soil properties are pivotal for determining water availability, nutrient uptake, and overall soil fertility, all of which are essential for the growth and vitality of *Eucalyptus* species (Lamoureux et al., 2018; Thurm et al., 2018; Reichert et al., 2023). Topographical factors, including elevation, slope, and aspect, provide key information on microclimatic variations, drainage patterns, and solar radiation exposure, all of which can significantly affect species establishment and survival (Ohmura, 2012). To account for anthropogenic influences, we included the Human Influence Index (HII), a comprehensive global dataset that synthesizes various human pressures, including population density, land use, infrastructure, and human accessibility. The HII provides a quantifiable measure of cumulative human impacts on natural environments, enabling us to identify areas where human activities may disrupt or modify *Eucalyptus* distributions (Gallardo et al., 2015; Frans and Liu, 2024). Topographical factors and soil factors were obtained from the Google Earth Engine (https://earthengine.google.com/). The human influence factor was obtained from the Socioeconomic Data and Applications Center (SEDAC) (https://sedac.ciesin.columbia.edu/).

For future climate modeling, we chose 2050s (2041-2060), 2070s (2061-2080). We used four Shared Socio-economic Pathways (SSPs): SSP126, SSP245, SSP370 and SSP585 can be made representing sustainability, middle of the road, regional rivalry and fossil-fueled development ways respectively. SSP126 assumes low emissions and significant mitigation efforts, with a global mean temperature increase of about 1.18 °C by 2100. SSP245 and SSP370 are intermediate scenarios with moderate emissions; SSP245 leads to a global mean temperature increase of about 3.22 °C, while SSP370 leads to a global mean temperature increase of about 5.50 °C. SSP585 reflects the highest emissions scenario, with a global mean temperature increase of about 7.20 °C by 2100 (Fan et al., 2020).

Future climatic projections were derived for four General Circulation Models (GCMs) developed within the Coupled Model Intercomparison Project Phase 6 (CMIP6): BCC-CSM2-MR, CMCC-ESM2, IPSL-CM6A-LR and MRI-ESM2-0. We averaged final model predictions for each SSP scenario across all four GCMs to account for uncertainty in future climate predictions related to the GCMs and to provide a more robust forecasts of range shifts (Goberville et al., 2015; Thuiller et al., 2019; Paź-Dyderska et al., 2021). All environmental factors were ultimately modeled and analyzed using ArcGIS 10.8 statistical resampling at a 2.5 arc-minutes resolution.

Identifying the environmental variables with erasing co-linearities among variables, requires the elimination of redundant variables (Dormann et al., 2013). An initial screening of the 27 environmental variables was carried out using Pearson’s correlation coefficient and jackknife. The retained environmental parameters if the Pearson’s correlation coefficient correlation coefficient was less than 0.8. If the coefficient exceeded 0.8, we eliminated the parameters with less ecological significance, based on the jackknife test results.

We finally retained 13 environmental factors for subsequent modelling, respectively were annual mean temperature (Bio1), mean diurnal range (Bio2), isothermality (Bio3), temperature seasonality (Bio4), temperature annual range (Bio7), mean temperature of coldest quarter (Bio11), annual precipitation (Bio12), precipitation of driest month (Bio14), precipitation of driest quarter (Bio17), precipitation of warmest quarter (Bio18), precipitation of coldest quarter (Bio19), human Influence Index (HII) and slope (**Table 1**).

**Table 1:** The final environmental factors for MaxEnt model and their contribution to predicting the distribution of *Eucalyptus grandis*, *E. urophylla*, and *E. tereticornis* in China.

Variables	Description	Percent contribution (%)		
		*E. grandis*	*E. urophylla*	*E. tereticornis*
Bio1	Annual mean temperature (°C)	11.6	5.6	33.3
Bio2	Mean diurnal range (°C)	–	8.3	–
Bio3	Isothermality (Bio2/Bio7) × 100	–	–	7.9
Bio4	Temperature seasonality (SD × 100)	–	–	32.6
Bio7	Temperature annual range (Bio5-Bio6) (°C)	7.6	–	–
Bio11	Mean temperature of coldest quarter (°C)	42.9	–	–
Bio12	Annual precipitation (mm)	3.1	30.3	11.8
Bio14	Precipitation of driest month (mm)	5.7	–	11.1
Bio17	Precipitation of driest quarter (mm)	–	5.6	–
Bio18	Precipitation of warmest quarter (mm)	17.8	36.6	–
Bio19	Precipitation of coldest quarter (mm)	–	–	2.7
Slope	Slope	–	12	0.5
HII	Human Influence Index	11.2	12.4	–

### 2.4 Data analysis

The MaxEnt model incorporates feature combinations (FCs) and regularization multipliers (RMs) to optimize the models and control overparameterization. Default settings of the MaxEnt model may lead to overfitting and biased prediction results (Elith et al., 2011). To mitigate this issue, we employed the ENMeval package to optimize the RMs and FCs of the MaxEnt model (Muscarella et al., 2014). This approach allowed us to evaluate model complexity and determine the optimal model parameters. RMs were varied from 0.5 to 4.0 with increments of 0.5. In the MaxEnt model, five FCs are available for selection: linear (L), quadratic (Q), hinge (H), product (P), and threshold (T). Adjusting these parameters can significantly enhance the model’s accuracy and stability (Phillips et al., 2006). Nine FCs were selected, including L, LH, LQ, LQH, LQHP, LQHPT, LQP, QHP and QHPT, and the “checkerboard2” method was employed to calculate the minimum Akaike information criterion coefficient (AICc), which reflects the model’s goodness of fit and complexity (Warren and Seifert, 2011). The combination of delta AICc values=0 was selected to run the best MaxEnt software among candidate models.

The MaxEnt model parameters used in this study are as follows: 75% of the occurrence data was randomly selected for training, while the remaining 25% was used for testing (Phillips et al., 2006). “Bootstrap” was chosen as the replicated run type and was replicated 10 times. The maximum iterations run 1000 times. Finally, the jackknife test and the contribution percentage were used to evaluate the importance of each environmental indicator, and create response curves to measure how the environmental variables affect each species. In this study, the Area Under the Curve (AUC) in Receiver Operating Characteristic (ROC) curve analysis and the True Skill Statistic (TSS) were used to assess the accuracy of the model results (Allouche et al., 2006; Peterson et al., 2008). ROC curves were constructed by using all possible thresholds to classify the scores into confusion matrices, obtaining sensitivity and specificity for each matrix, and then plotting sensitivity against the corresponding proportion of false positives. AUC values range from 0 to 1, where 1 indicates a perfect fit, and 0.5 indicates that predictions from the SDM do not differ from random, and 0 means the SDM is always incorrect (van Proosdij et al., 2016). TSS is a threshold-dependent metric calculated as: sensitivity +specificity -1. TSS ranges from -1 to 1, where 1 indicates perfect agreement, 0 indicates a random prediction and negative values indicate that predictions perform worse than random. The MaxEnt output contains suitability maps with continuous values between 0 (unsuitable) and 1 (highly suitable). We converted the species continuous distribution model into a threshold-based binary map, where we used the value with the highest sum of sensitivity (true positive rate) and specificity (true negative rate) as the presence threshold (Puchałka et al., 2023). To further refine the suitable range changes for each species under different future climate scenarios, we compared potential future distribution areas with current potential distributions in ArcGIS using SDMtoolbox. We categorized the results into three categories: expanding, stabilizing, and disappearing. To calculate the impact of the climate scenarios on predicted potential distribution, we measured the percent change in potential distribution areas.

## 3 Results

### 3.1 Model optimization and its accuracy

The default parameter settings for the Maxent model are RM=1 and FC=LQHPT. We optimized these settings using the ENMeval package in R, and the optimal parameters for the three species are presented in **Table 2**. According to Akaike’s information criterion, the model with the smallest AICc value (delta AICc=0) demonstrated the lowest complexity, suggesting minimal overfitting. Using these optimal parameters, we applied the Maxent model to predict potentially suitable areas for three *Eucalyptus* species and calculated the AUC and TSS values for each. The AUC values for *E. grandis*, *E. urophylla*, and *E. tereticornis* were 0.973, 0.983, and 0.901, respectively, while the TSS values were 0.928, 0.941, and 0.923, respectively (**Table 3**). These results indicate model performance and high reliability.

**Table 2:** Evaluation results of MaxEnt model under different parameter setting of *Eucalyptus grandis*, *E. urophylla*, and *E. tereticornis*.

*E. grandis*			
Setting	FC	RM	delta.AICc
Default	LQHPT	1	1.569723
Optimized	LQHPT	1.5	0
*E. urophylla*			
Setting	FC	RM	delta.AICc
Default	LQPHT	1	198.6067
Optimized	LQH	0.5	0
*E. tereticornis*			
Setting	FC	RM	delta.AICc
Default	LQHPT	1	161.6846
Optimized	LQHPT	0.5	0

Delta Akaike information criterion coefficient (delta.AICc). The MaxEnt model incorporates feature combinations (FCs) and regularization multipliers to optimize the models and prevent overfitting. In the MaxEnt model, five FCs are available for selection: linear (L), quadratic (Q), hinge (H), product (P), and threshold (T). Adjusting these parameters may significantly enhance the model’s accuracy and stability. The combination of delta.AICc values = 0 was selected to run the best MaxEnt software among candidate models.

**Table 3:** Studied species, number of data points, and model characteristics.

Species	Number of input points	Points after screening	AUC	TSS	Probability occurrence threshold
*E. grandis*	2379	344	0.973	0.928	0.149
*E. urophylla*	352	217	0.983	0.941	0.167
*E. tereticornis*	20,503	2645	0.901	0.923	0.218

### 3.2 Important environmental variables

**Figure 2:**
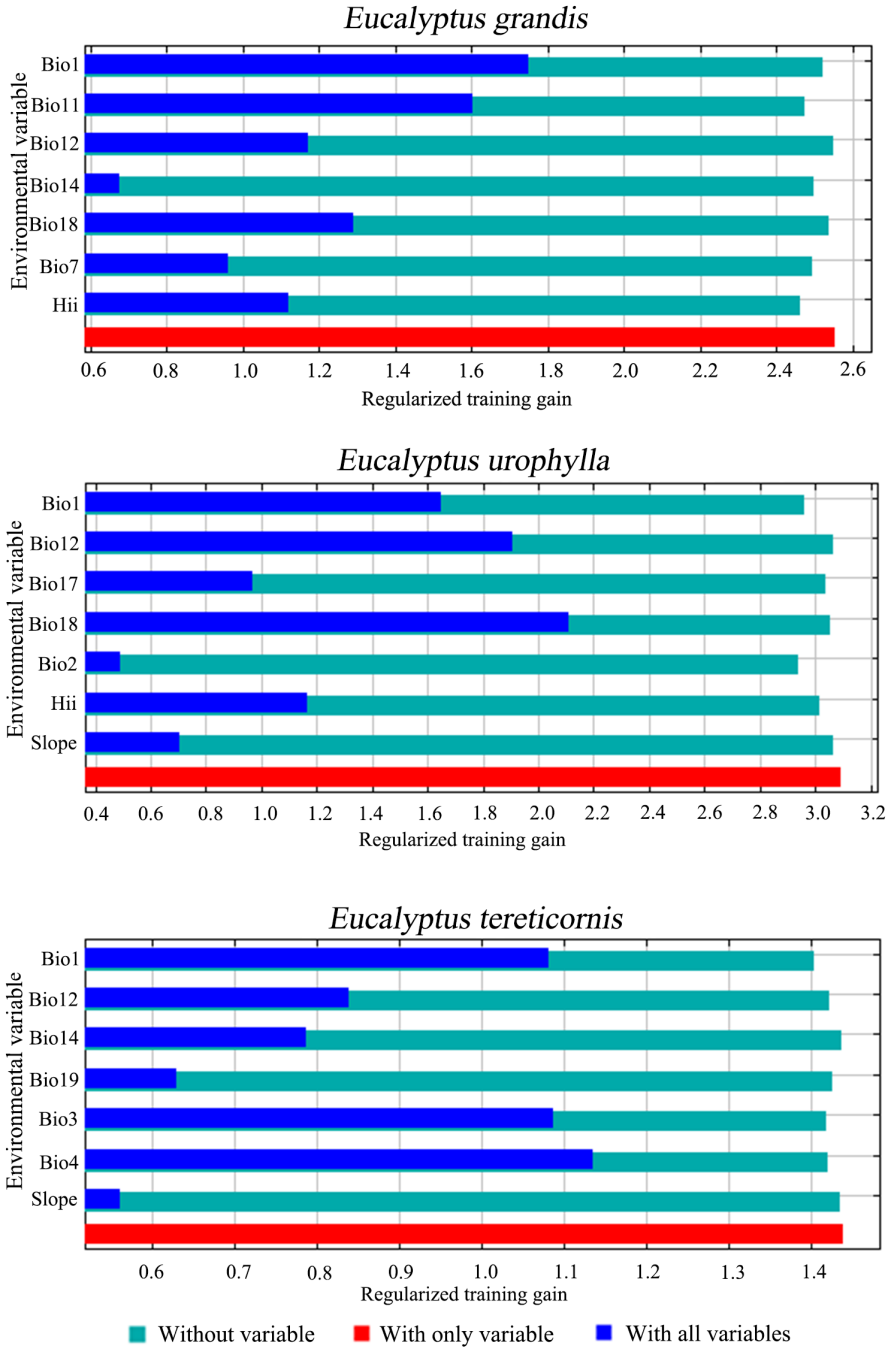
Jackknife test of important environment variables for three *Eucalyptus* species.

The jackknife test results and percentage contribution can confirm the primary environmental variables that affect potential distribution areas (**Table 1**; **Figure 2**). The results indicated that the main environmental variables affecting *E. grandis* were mean temperature of the coldest quarter (Bio11, 42.9%), precipitation of the warmest quarter (Bio18, 17.8%), annual mean temperature (Bio1, 11.6%), and human influence index (HII, 11.2%). Precipitation of the warmest quarter (Bio18, 36.6%) emerged as the most influential variable for the distribution of *E. urophylla*, followed by annual precipitation (Bio12, 30.3%), Human influence index (HII, 12.4%), and mean diurnal temperature range (Bio2, 8.3%). For *E. tereticornis*, annual mean temperature (Bio1, 33.3%) contributed the most, followed by temperature seasonality (Bio4, 32.6%), annual precipitation (Bio12, 11.8%), and precipitation of the driest month (Bio14, 11.1%). The cumulative contribution of these environmental variables to each *Eucalyptus* species reached values as high as 80% or even higher.

**Figure 3:**
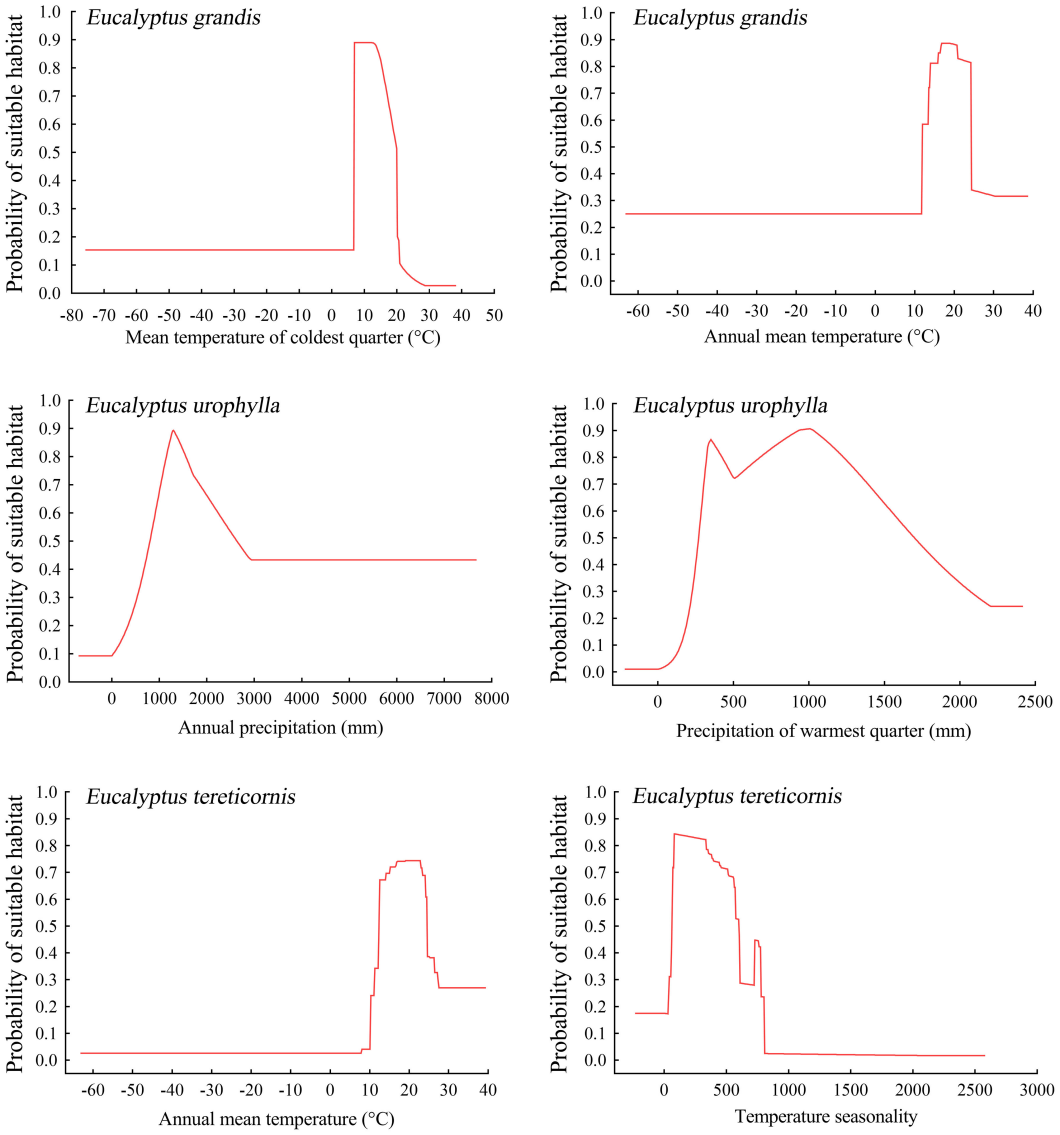
Prediction of habitat suitability for three *Eucalyptus* species by different environmental variables.

The Maxent model offers response curves to elucidate the relationship between the probability of species presence and environmental factors, aiding in understanding how each factor affects habitat distribution. It is generally accepted that when the habitat suitability exceeds 0.5, the corresponding environmental factor value is considered suitable for the species. Below are the response curves for the two most influential climatic factors affecting the current distribution of potentially suitable areas for the three *Eucalyptus* species. According to the results of the response curves, *E. grandis* exhibited optimal growth conditions when the mean temperature of the coldest quarter ranged from 7.0 to 20.0 °C, and the annual mean temperature ranged from 11.9 to 24.2 °C. *Eucalyptus urophylla* demonstrated the highest suitability for growth when precipitation of the warmest quarter ranged from 272 to 1694 mm, and annual precipitation ranged from 812 to 2624 mm. Additionally, annual mean temperature ranging from 12.7 to 24.5 °C and temperature seasonality ranging from 63.8 to 598.9 were identified as most suitable for *E. tereticornis* (**Figure 3**).

### 3.3 Potential distribution of three *Eucalyptus* species under the current climate

**Figure 4:**
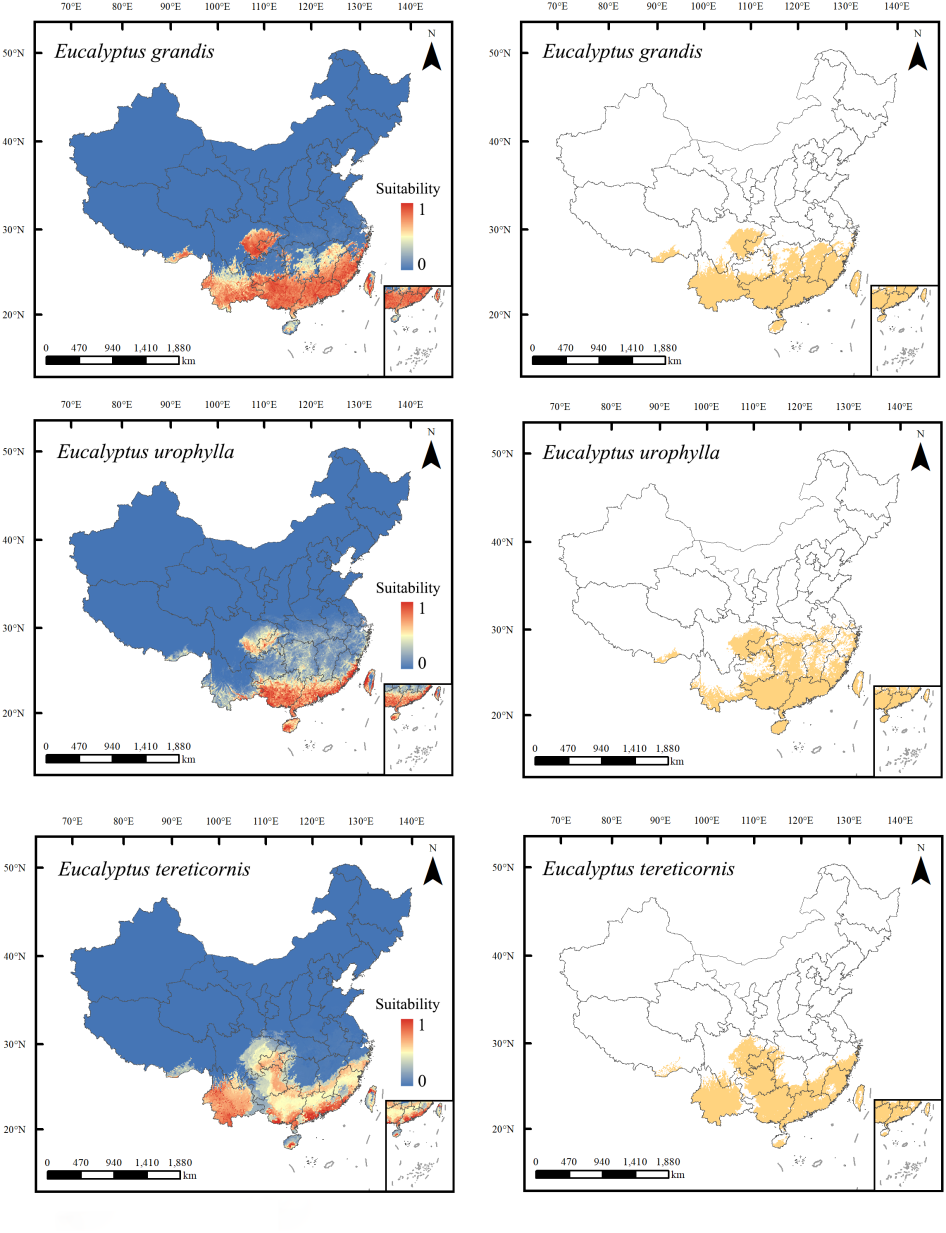
Predicted suitable habitats for *Eucalyptus grandis, E. urophylla*, and *E. tereticornis* under current climate conditions and land use change within their range. The left and right panels of the range maps show continuous and binary suitability scores, respectively. The binary maps were generated using a threshold that produced the highest sum of sensitivity and specificity.

These three species exhibit distinct differences in their current potential suitability areas (**Figure 4**). *Eucalyptus tereticornis*, the most widely distributed species in this study, occupies a total potential suitable area of 124.91 × 10^4^ km², distributed across Guangdong, Guangxi, Yunnan, Fujian, Hainan, Taiwan, Guizhou, and Chongqing, along with portions of eastern Sichuan, southern Hunan, southern Jiangxi, southern Zhejiang, and southeast Tibet. The total potentially suitable area of *E. grandis* is 124.89 × 10^4^ km², covering regions in Guangxi, Guangdong, Hainan, Yunan, Taiwan, Fujian, Jiangxi, along with portions of eastern Sichuan, western Chongqing, southern Guizhou, southern Zhejiang, central and eastern parts of Hunan, and southeast Tibet. *Eucalyptus urophylla* has a total potential suitable area of 119.81 × 10^4^ km^2^, mainly in Guangxi, Guangdong, Hainan, Hunan, Chongqing, Taiwan, eastern Fujian, southern Yunnan, eastern Sichuan, southern Anhui, southern Hubei, central and southern Jiangxi, central and eastern Zhejiang, and southeast Tibet.

### 3.4 Changes in the potential distribution of three *Eucalyptus* species under future climate change

**Figure 5:**
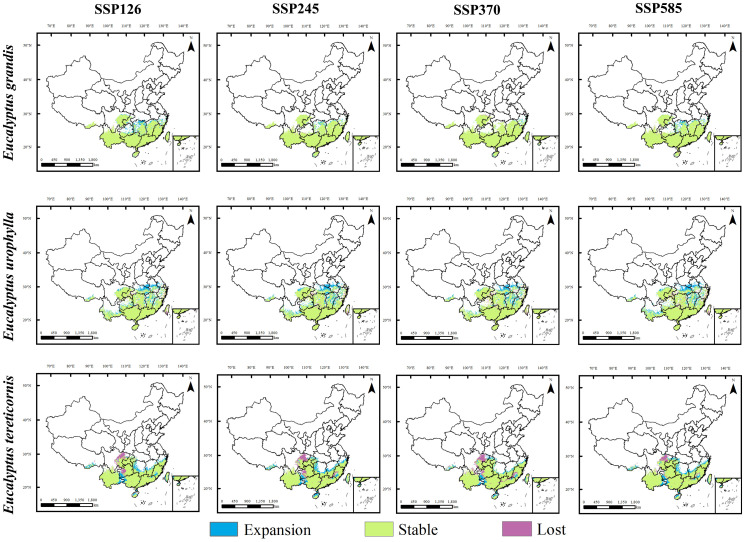
Regional differences in the future and current potential distribution of three *Eucalyptus* species under four climate change scenarios in 2050.

**Figure 6:**
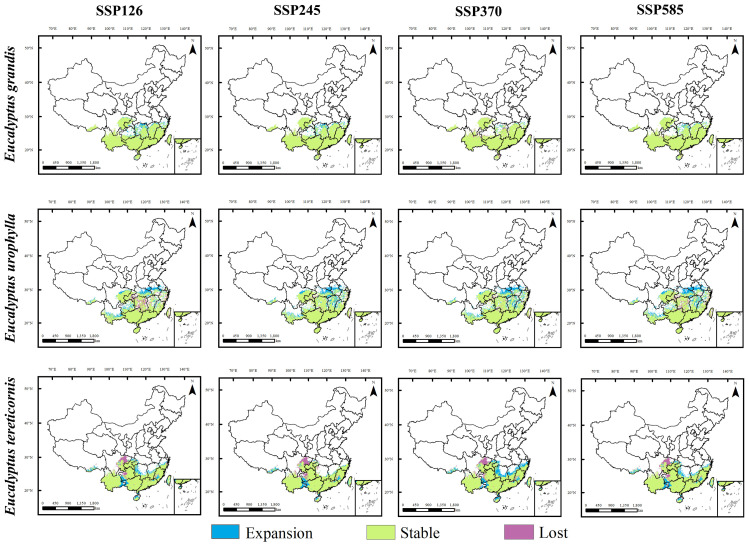
Regional differences in the future and current potential distribution of three *Eucalyptus* species under four climate change scenarios in 2070.

We projected the response of *E. grandis*, *E. urophylla* and *E. tereticornis* to climate change in 2050s and 2070s under future climate scenarios by comparing them with current potential habitat areas. Potential habitat areas for these three species showed different trends in 2050s and 2070s (**Figures 5**, **6**).

Under future climatic conditions, the suitable area of *E. grandis* will remain stable with a slight increase, and its expansion range will expand slightly mainly to the north, with new expansion areas occurring mainly in the northern part of Hunan Province, the northern part of Jiangxi Province, and the southern part of Zhejiang Province. Unlike *E. grandis*, the projected suitable area for *E. urophylla* experiences significant expansion and slight contraction, with expansion mainly to the north of the suitable area, with the largest area of expansion under the 2070s SSP585 scenario. The areas of expansion were mainly located in northern Jiangxi, northern Hubei, central Anhui, central Zhejiang and southern Henan provinces, and the areas of contraction were mainly located in central Hunan and southern Chongqing. For *E. tereticornis*, the future suitable area expanded in the northern part of its current range. Under the future climate scenarios, its suitable area shrinks mainly in eastern Sichuan and western Guizhou, with the greatest loss and least expansion under the 2070s SSP245 scenario and the greatest expansion under the 2070s SSP370 scenario.

**Figure 7:**
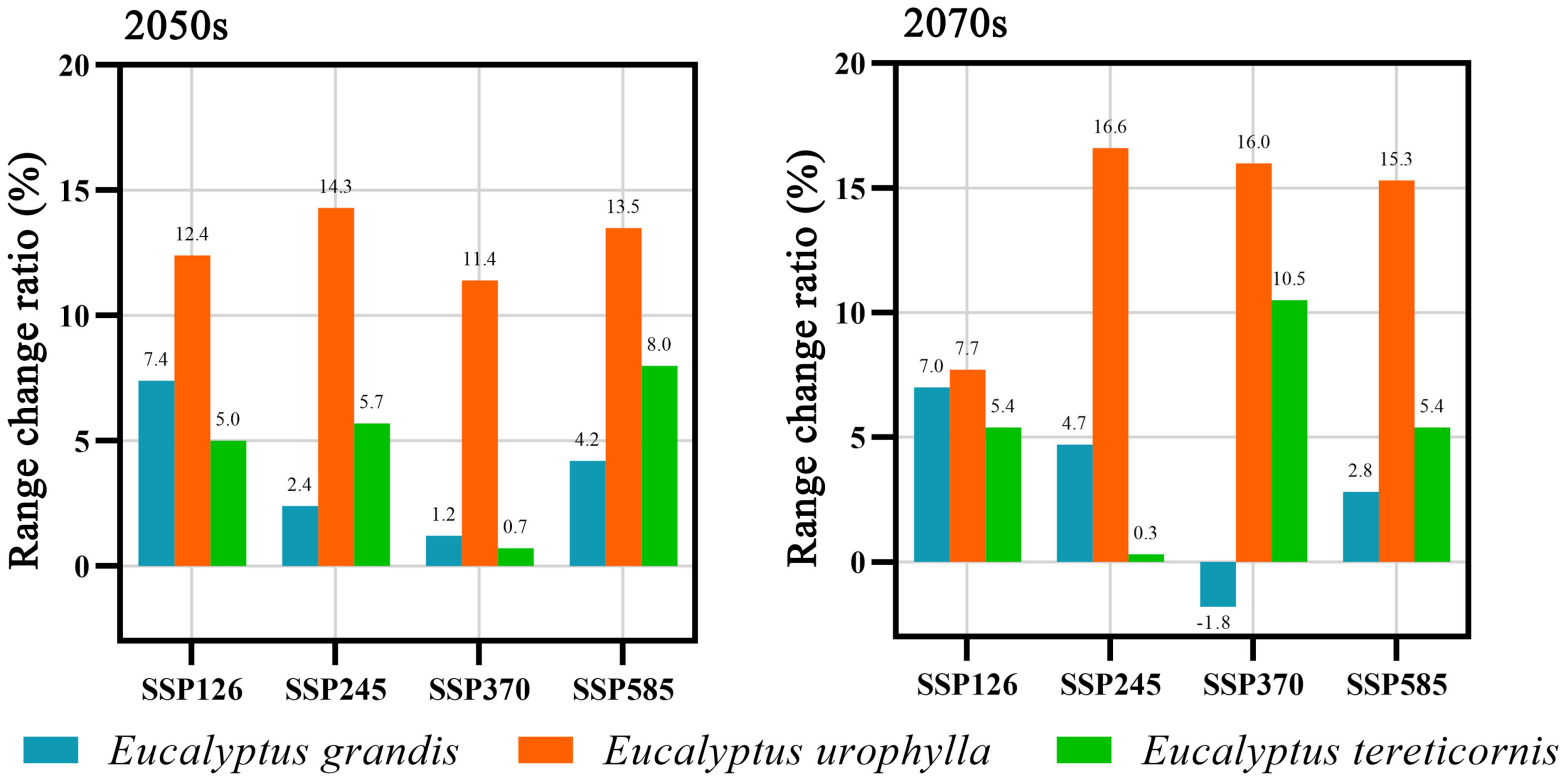
Proportion of range shifts of suitable areas for three *Eucalyptus* species under four climate change scenarios and two timelines.

Under different climate scenarios and timelines, except for a reduction of 1.8% in the distribution area of *E. grandis* under the SSP370 scenario in 2070s, the distribution areas of all species under other future climate scenarios have expanded (**Figure 7**). Among them, the suitable area of *E. urophylla* increases the most, with a change rate ranging from 7.7% to 16.6%. The change trends of *E. grandis* and *E. tereticornis* are similar, with a smaller increase in potential suitable area. The change rate of *E. grandis* ranges from 1.2% to 7.4%, while that of *E. tereticornis* ranges from 0.7% to 10.5%.

## 4 Discussion

In this study, we employed the MaxEnt model to predict the current and future potential suitable distribution of three *Eucalyptus* species in China. Our results demonstrate that the performance of the MaxEnt model achieved a high level of accuracy (AUC and TSS values exceeding 0.9), indicating the model’s reliability and precision in predicting suitable areas. Therefore, we believe that the performance of our model is robust enough to account for the overall distribution of suitable areas for three *Eucalyptus* species in China. The results for the three *Eucalyptus* species confirm our expectation that most of the predicted changes in potential range from 2061-2080 can be observed 20 years ahead. We demonstrated that *E. tereticornis* has the largest potential suitable area, and that the potential suitable area for all three species will expand under future climatic conditions.

### 4.1 Potential distribution of three *Eucalyptus* species under the current environment

The MaxEnt modeling results revealed that annual precipitation and annual mean temperature were consistent key environmental factors influencing the distribution of *E. grandis*, *E. urophylla*, and *E. tereticornis* (**Table 1**). Consequently, we consider precipitation and temperature as essential determinants for the survival and distribution of *Eucalyptus* species. However, when other factors were considered, *E. grandis*, *E. urophylla* and *E. tereticornis* differed significantly in temperature seasonality, precipitation of warmest quarter and mean temperature of coldest quarter differed significantly in their importance.

The mean temperature of coldest quarter (7.0-20.0 °C) and annual mean temperature (11.94-24.2 °C) are the most crucial environmental factors influencing the distribution of suitable areas for *E. grandis*. This finding aligns with the known habitat preferences of *E. grandis*, which favors warm and humid climates while being vulnerable to extreme cold and high temperatures (Liu et al., 2023). Our study’s results corroborate these observations, highlighting the significance of temperature parameters in shaping the suitability of areas for *E. grandis*. Annual mean temperature (12.7-24.5 °C) and temperature seasonality (63.8-598.9) emerge as the most critical environmental factors influencing the distribution of suitable areas for *E. tereticornis*. Studies have indicated that *E. tereticornis* exhibits excellent growth in areas with an annual mean temperature of approximately 14 °C (Booth, 2019). Precipitation of warmest quarter (272-1694 mm) and annual precipitation (812-2624 mm) were the most critical environmental factors for *E. urophylla* (**Table 1**). In Brazil, *E. urophylla* does not thrive in semi-arid regions with annual precipitation less than 800 mm, precipitation is a prerequisite for the distribution of suitable areas for *E. urophylla* (Scanavacca Júnior and Garcia, 2021).

*Eucalyptus* species exhibit a preference for warm temperatures and have relatively low tolerance to cold. Based on surveyed sample plots, the latitude range of their distribution in plantation forests typically falls within 18°18′ to 31°3′ N. This observation generally aligns with our findings indicating that the potential distribution of *E. tereticornis* ranges from approximately 18°10′ to 31°3′ N. latitude (Zhang and Wang, 2021). Among these species, *E. tereticornis* demonstrates the widest distribution of suitable areas, followed by *E. grandis*, and finally *E. urophylla*. In January 2003, Hengyang, Hunan Province experienced its lowest temperature recorded at -7 °C. Subsequently, all *E. urophylla* in Hengyang died, and some *E. grandis* was severely damaged. Similarly, in Fujian, where there are over 2,000 hectares of *E. urophylla* and *E. urophylla* × *E grandis* plantations at high altitudes, severe winter cold (-8 °C) in December 1999 led to the death or severe damage of *E. urophylla* and *E. urophylla* × *E. grandis* (Arnold and Luo, 2004). These incidents occurred in areas located at the junction of our predicted suitable and unsuitable areas. We hypothesize that these phenomena may be attributed to the reduced suitability of *E. grandis* and *E. urophylla* under the climatic conditions of marginal production areas. If the climatic conditions fall outside the suitable range for *Eucalyptus* growth, it may result in extensive *Eucalyptus* mortality. In such cases, we do not recommend large-scale *Eucalyptus* planting activities at the junction of our predicted suitable and unsuitable areas.

Based on the MaxEnt results of this study, we propose that *Eucalyptus* plantations can be established and cultivated in coastal areas of Guangdong, Guangxi, Yunnan, eastern Sichuan, and Fujian in China. We recommend that planting activities be carried out mainly in areas of high suitability. Among the three *Eucalyptus* species studied, *E. tereticornis* exhibits the largest area of high suitable areas, totaling 124.91 × 10^4^ km². In comparison with the current national *Eucalyptus* plantation area, which stands at 8.95 × 10^4^ km^2^, there remains significant potential for the development and promotion of *Eucalyptus* plantation expansion.

### 4.2 Potential distribution change of *Eucalyptus* species under future climate change

The findings of this study highlight significant differences in ecological habits among *E. grandis*, *E. urophylla*, and *E. tereticornis*. It is anticipated that they will exhibit markedly distinct responses in the face of future climate change. It is well demonstrated that the species responses to climate change vary with species and regions. In Mexico, climate change could lead to a reduction in potential future suitable areas for *Swietenia macrophylla* and *Cedrela odorata* (Hernández Ramos et al., 2018; Ramírez-Magil et al., 2020). In Europe, alien coniferous species will contract and deciduous trees will expand their climatic niche under climate change scenarios (Puchałka et al., 2023). Under the SSP370 scenario for the period 2061-2080, the ranges of *E. grandis* and *E. urophylla* are projected to increase, while the range of *E. tereticornis* is projected to decrease. Different species have different tolerances and preferences for climate change (Hamann et al., 2021b).

Numerous studies have shown that climate warming is causing many species to migrate to higher latitudes in search of suitable climatic ecological niches (Chen et al., 2011; Giesecke et al., 2019; Feng et al., 2021). In our study, *E. grandis*, *E. urophylla*, and *E. tereticornis* were observed to exhibit a similar trend of northward movement. This pattern could be attributed to the northward shift of climate zones driven by climate warming, leading to the emergence of more suitable areas at higher latitudes (Liu et al., 2023). Our study found that *E. tereticornis* demonstrated significant fragmentation of suitable areas under different emission pathways. This trend resembles findings from previous studies on C. lanceolata in China and may be associated with the more frequent predictions of extreme temperatures expected in China under future climatic conditions (Zhao et al., 2021b; Yu et al., 2023).

Based on the projected shifts in the suitable habitats of *E. grandis*, *E. urophylla*, and *E. tereticornis* under future climate scenarios, strategic adjustments should be made to *Eucalyptus* planting in China to maximize productivity and sustainability. Under future climatic conditions, caution should be exercised in planting *Eucalyptus* species in the contraction areas, including *E. urophylla* (Hunan, central Jiangxi and southern Chongqing), and *E. tereicornis* (northern Guangdong, eastern Sichuan, and western Guizhou). In contrast, Yunnan, Guangdong, Guangxi, Fujian, Jiangxi, Hainan and Taiwan in China, which will be relatively less affected by climate change, could be used as a base for large-scale cultivation, and utilization of *E. grandis* in the future. The Hainan, Guangdong, and Guangxi, Fujian, can serve as a cultivation base for *E. urophylla*, and the Yunnan, Guangxi, and Hainan can be used as a cultivation base for *E. tereicornis*. This study offers valuable insights into the future introduction and cultivation of *Eucalyptus* species. It is imperative to carefully consider the future changes in the distribution extent of different *Eucalyptus* species under various climate scenarios when planning and implementing *Eucalyptus* plantations. Additionally, planting efforts should integrate broader ecological and socioeconomic considerations, including water availability and local ecosystem impacts, to ensure that expansion does not exacerbate existing environmental challenges.

### 4.3 Limitations of this study

First, this study used only the MaxEnt model to simulate and predict *E. grandis*, *E. urophylla*, and *E. tereticornis* potential distributions. Previous studies have found that the prediction results of an ensemble model will outperform a poorly performing single model and underperform a better performing single model (Zhu and Peterson, 2017). Secondly, the data used for species distribution modeling can also be influenced by observer bias and uneven sampling, as public repositories frequently contain datasets with varying coverage and quality (Cornwell et al., 2019). Third, projections based on GCMs introduce uncertainty due to the inherent variability in future climate scenarios. While averaging across multiple GCMs enhances reliability, it does not eliminate the uncertainties linked to model variability (Thuiller et al., 2019; Paź-Dyderska et al., 2021).

In addition, although the model takes into account climate, soil and topography, and HII influences, it does not add to the predictions because future soil, topography, and HII data are difficult to obtain. The increasing interest in *Eucalyptus* cultivation has led to annual reductions in arable land, as large areas are converted to *Eucalyptus* plantations. Human activities have substantially modified land use patterns, leading to shifts in ecosystem functioning and taxonomic diversity (Cardinale et al., 2012). Extensive research has underscored the adverse environmental effects and invasive characteristics linked to large-scale *Eucalyptus* plantations, which can significantly impact native crop species, trees, and animal populations (Mengistu et al., 2022; Randriamalala et al., 2023; Tesfaw et al., 2023). Future research should consider biological interactions and their integration into species distribution models. The potentially suitable areas obtained in this study have limitations. In practical applications, local soil and hydrogeologic conditions must be integrated. However, these study results represent the initial stage of macro-planning and serve as a crucial guide for rational *Eucalyptus* cultivation.

## 5 Conclusions

In this study, we employed an optimized MaxEnt model to explore the impact of climate, topography, soil, and human influence index data on the potential distribution of three *Eucalyptus* species. Our findings revealed substantial ecological disparities among *Eucalyptus grandis*, *Eucalyptus urophylla*, and *Eucalyptus tereticornis* in China. The potential distribution of *E. grandis* and *E. tereticornis* is primarily influenced by temperature, whereas precipitation predominantly shapes the potential distribution of *E. urophylla*. Our results suggest that future climate change leads to an expansion of potentially suitable areas for *E. tereticornis*, *E. grandis* and *E. urophylla*. The results of this study hold significant implications for ensuring the stability of plantation forest ecosystems in China, offering a scientific foundation for the planning and management of *Eucalyptus* species.

## Data Availability

The original contributions presented in the study are included in the article/**Supplementary Material**. Further inquiries can be directed to the corresponding author.
