# Supplementary material for: Climate change favors expansion of three Eucalyptus species in China
Source: Front Plant Sci. 2024 Oct 11;15:1443134. doi: 10.3389/fpls.2024.1443134 (PMC11502323; doi:10.3389/fpls.2024.1443134)
Supplement: Supplementary file 1 [file Table1.docx]

**Supplementary Material**

**Climate change favors expansion of three *Eucalyptus* species in China**

Xinjie Mao, Huisen Zheng, Guihua Luo, Songkai Liao, Ronghao Wang, Ming Tang, Hui Chen^*^

* **Correspondence:** Hui Chen: chenhui@scau.edu.cn

**Table S1** The names of the 27 environment variables in the research.

| Category | Abbreviations | Description |
| --- | --- | --- |
| Climate | Bio1 | Annual Mean Temperature (℃) |
|  | Bio2 | Mean Diurnal Range (℃) |
|  | Bio3 | Isothermality (Bio2/Bio7) × 100 |
|  | Bio4 | Temperature Seasonality (SD × 100) |
|  | Bio5 | Max Temperature of Warmest Month (℃) |
|  | Bio6 | Min Temperature of Coldest Month (℃) |
|  | Bio7 | Temperature Annual Range (Bio5-Bio6) (℃) |
|  | Bio8 | Mean Temperature of Wettest Quarter (℃) |
|  | Bio9 | Mean Temperature of Driest Quarter (℃) |
|  | Bio10 | Mean Temperature of Warmest Quarter (℃) |
|  | Bio11 | Mean Temperature of Coldest Quarter (℃) |
|  | Bio12 | Annual Precipitation(mm) |
|  | Bio13 | Precipitation of Wettest Month(mm) |
|  | Bio14 | Precipitation of Driest Month(mm) |
|  | Bio15 | Precipitation Seasonality (Coefficient of Variation) |
|  | Bio16 | Precipitation of Wettest Quarter(mm) |
|  | Bio17 | Precipitation of Driest Quarter(mm) |
|  | Bio18 | Precipitation of Warmest Quarter(mm) |
|  | Bio19 | Precipitation of Coldest Quarter(mm) |
| Terrain | Dem | Digital Elevation Model |
|  | Slope | Slope |
|  | Aspect | Aspect |
| Environment | HII | Human Influence Index |
| Soil quality | Sand | Proportion of sand particles(>0.05mm) in the fine earth fraction |
|  | Clay | Proportion of clay particles(<0.002mm) in the fine earth fraction |
|  | Silt | Proportion of silt particles (0.002-0.05mm) in the fine earth fraction |
|  | pH | Soil pH |
